# Hypothalamic Neurochemical Changes in Long-Term Recovered Bilateral Subdiaphragmatic Vagotomized Rats

**DOI:** 10.3389/fnbeh.2022.869526

**Published:** 2022-07-08

**Authors:** Anna Teresa Kobrzycka, Adrian Mateusz Stankiewicz, Joanna Goscik, Monika Gora, Beata Burzynska, Roksana Iwanicka-Nowicka, Krystyna Pierzchala-Koziec, Marek Wieczorek

**Affiliations:** ^1^Department of Neurobiology, Faculty of Biology and Environmental Protection, University of Łodz, Łodz, Poland; ^2^Department of Molecular Biology, Institute of Genetics and Animal Biotechnology, Polish Academy of Sciences, Jastrzebiec, Poland; ^3^Software Department, Faculty of Computer Science, Bialystok University of Technology, Bialystok, Poland; ^4^Department of Genetics, Institute of Biochemistry and Biophysics, Polish Academy of Sciences, Warsaw, Poland; ^5^Faculty of Biology, University of Warsaw, Warsaw, Poland; ^6^Department of Animal Physiology and Endocrinology, University of Agriculture in Krakow, Krakow, Poland

**Keywords:** hypothalamus, subdiaphragmatic vagotomy, amino acid neurotransmitters, inflammatory response, microarray, HPA axis

## Abstract

**Background:**

Vagus nerve is one of the crucial routes in communication between the immune and central nervous systems. The impaired vagal nerve function may intensify peripheral inflammatory processes. This effect subsides along with prolonged recovery after permanent nerve injury. One of the results of such compensation is a normalized plasma concentration of stress hormone corticosterone – a marker of hypothalamic-pituitary-adrenal (HPA) axis activity. In this work, we strive to explain this corticosterone normalization by studying the mechanisms responsible for compensation-related neurochemical alterations in the hypothalamus.

**Materials and Methods:**

Using microarrays and high performance liquid chromatography (HPLC), we measured genome-wide gene expression and major amino acid neurotransmitters content in the hypothalamus of bilaterally vagotomized rats, 1 month after surgery.

**Results:**

Our results show that, in the long term, vagotomy affects hypothalamic amino acids concentration but not mRNA expression of tested genes.

**Discussion:**

We propose an alternative pathway of immune to CNS communication after vagotomy, leading to activation of the HPA axis, by influencing central amino acids and subsequent monoaminergic neurotransmission.

## Introduction

The vagus nerve, due to its extensive peripheral projection range, is a prominent interoceptive pathway (Forsythe et al., 2014). The vagus nerve transmits intraperitoneal immune signals to the CNS (Watkins and Maier, 2005; Quan and Banks, 2007; Dantzer, 2009; D’Mello and Swain, 2016), causing neurotransmission alterations (Wieczorek et al., 2005; Dunn, 2006; Hopkins, 2007), behavioral changes (Wieczorek et al., 2005; Dantzer and Kelley, 2007; Dantzer et al., 2008) and indirect activation of the hypothalamus (Wieczorek and Dunn, 2006; Noori et al., 2017) a first element of the hypothalamic-pituitary-adrenal (HPA) axis of stress. HPA activation results in secretion of glucocorticoids (GCs), which are important regulators of the immune system (Hosoi et al., 2000). Moreover, the parasympathetic part of the vagus nerve can directly influence the activity of immune cells in internal organs exposed to pathogens through the cholinergic anti-inflammatory pathway (Tracey, 2007; Karimi et al., 2010; Pereira and Leite, 2016).

The physiological tonic activity of the vagus nerve (so-called vagal tone) is crucial for maintaining body allostasis. Vagal tone is a critical regulator of the inflammatory response. Low vagal tone results in increased basal plasmatic cortisol, tumor necrosis factor α (TNF-α), and epinephrine levels, which Bonaz et al. (2016) summarized as a pro-inflammatory effect (Pellissier et al., 2014; Bonaz et al., 2016). Vagal tone is also involved in regulating emotional and behavioral responsiveness as well as sickness behaviors – a natural response to ongoing inflammation, which supports the immune system in its fight against the pathogen (Porges et al., 1994; Porges, 1995). The experimental model of abolished vagal tone in context of peritoneal vagal functions is a full subdiaphragmatic truncal vagotomy, which we used in the current experiment.

Vagus nerve integrity is necessary for proper inhibition of inflammatory response. Thus, vagotomy results in highly intensified inflammation and decreased secretion of anti-inflammatory GCs. Surprisingly, these effects diminish after a prolonged period of recovery following vagotomy (Ghia et al., 2007; Mitsui et al., 2014).

Previously, we reported that 30 days after subdiaphragmatic vagotomy LPS-induced increase of plasma corticosterone is similar to the control group (Kobrzycka et al., 2019). At the same time, we reported that monoaminergic neurotransmission is altered in the hypothalamus after a prolonged period of recovery following vagotomy, which might contribute to the restoration of HPA axis activity. These neurotransmission changes may occur due to the presence of compensatory mechanisms for impaired immune sensory and anti-inflammatory functions of the vagus nerve. Wieczorek and Dunn (2006) suggested that function of the damaged vagal nerve is substituted by COX-dependent pathway, where immune signals are carried by blood-borne prostaglandin E2 (PGE2). Corroborating this hypothesis, we previously observed that the concentration of PGE2 under inflammatory conditions is increased in the plasma of vagotomized animals, 30 days after vagotomy procedure (Kobrzycka et al., 2019).

In this work, we present a data set investigated simultaneously with those presented previously (Kobrzycka et al., 2019). Here, we investigate whether transcriptomic changes and/or altered amino acid neurotransmission accompanies monoaminergic alterations in the hypothalamus and, in consequence, may be responsible for the preservation of HPA axis activity after recovery from vagotomy. To test those hypotheses, we performed an RNA microarray with subsequent RT-qPCR analysis and high performance liquid chromatography (HPLC) concentration analysis of excitatory and inhibitory amino acid neurotransmitters.

## Materials and Methods

The studies were performed on 3-month-old male Wistar rats (300 g ± 25 g, *N* = 75). Animals were individually housed in breeding cages under the following conditions: free access to water and feed (Purina granules), artificial lighting conditions (a 12-h day-night cycle, light on at 7.00 AM), temperature 21–22°C and 60–65% humidity. Before the start of the experiments, the animals were habituated for 7 days to the conditions in the animal facility.

### Experiment

The animals were divided into two main groups: sham operated (SH, *n* = 31) and subdiaphragmatically vagotomized (VG, *n* = 33). Animals in the VG group were subjected to surgery under general anesthesia, induced by an intraperitoneal injection of Innovar plus (6 μl/g body weight) and local anesthesia with 2% lidocaine solution (0.5 ml/animal). During operation, a small fragment of gastrointestinal and hepatic branches of the vagal nerve was cut just below the diaphragm. To prevent post-operative infection and to facilitate wound healing, an antibacterial agent (Alu Spray, V.M.D.) was applied to the operation site. Bilateral subdiaphragmatic truncal vagotomy procedure and its validation are described in detail in Kobrzycka et al. (2019). Sham operation (SH) was performed in an analogous way to subdiaphragmatic vagotomy, with exception of cutting the nerves. Following surgeries, the animals were returned to their cages, and after a 30-day-long recovery period, the animals from SH and VG groups were intraperitoneally injected with either saline (0.9% NaCl, 100 μl i.p., SH *n* = 15, VG *n* = 15) or LPS (10 μg *E. coli* 026:B6 in 100 μl 0.9% NaCl, 100 μl i.p., SH *n* = 16, VG *n* = 18). After 120 min, the animals were decapitated.

Immediately after decapitation, hypothalamus (HPT) samples, restricted mainly to the medial part containing the paraventricular nucleus (PVN), were taken. Immediately after decapitation, the brain was cut on a glass plate kept on ice. After the section, samples assigned for HPLC analysis were placed in tubes on dry ice, weighed, and processed in homogenization buffer – the whole procedure took up to 5 min. The samples assigned for RNA analysis were placed in RNA-later solution right after dissection (within 2–3 min after decapitation). HPT samples weighted on average 0.05 g (±0.02 g). Stereotactic coordinates used to locate HPT were: AP = −1.44 to −2.04, *L* = ±1.5, *H* = −8 to −10 according to the Paxinos and Watson stereotaxic atlas (Paxinos and Watson, 1998).

### Samples Preparation Procedures

For the enzyme-linked immunosorbent assay (ELISA) test of pro-inflammatory cytokines, plasma was obtained by centrifuging the trunk blood collected on EDTA (1 ml/100 μl of Na_2_ EDTA), 4,000 rpm for 10 min at 4°C. The obtained plasma was frozen and stored at −80°C until future analysis. For HPLC analysis of amino acid neurotransmitters, hypothalamuses (SH NaCl *n* = 6, VG NaCl *n* = 7, SH LPS *n* = 7, VG LPS *n* = 8) were immediately homogenized using an ultrasonic homogenizer (Fisher BioBlock Scientific, France) for 15 s in 150 μl homogenization solution (0.4 mM Na_2_S_2_O_5_, 0.6 mM HClO_4_), and centrifuged at 12,000 rpm for 15 min at 4°C. At least 100 μl of the supernatant was collected from each sample, transferred to chromatographic tubes and frozen at −80°C.

For microarray and PCR analyses, hypothalamuses (naive control CNT *n* = 11, SH NaCl *n* = 9, VG NaCl *n* = 8, SH LPS *n* = 9, VG LPS *n* = 10) were stored in −20°C in 200 μl of RNA-later (Thermo Fisher Scientific, United States). Total RNA was extracted from dissected hypothalamuses using a spin column-based Universal RNA Purification Kit (EURx, Poland) according to the manufacturer’s protocol. RNA samples were subjected to DNase treatment on-column (RNase-free DNase I, EURx, Poland, 15 min, room temperature) as well as in-sample (TURBO DNA-free Kit, Thermo Fisher Scientific, United States, 30 min, 37°C), to remove any residual DNA contamination. The quantity, purity, and quality of RNA samples were estimated using a NanoDrop spectrophotometer (Thermo Fisher Scientific, United States) and a Bioanalyzer 2100 microcapillary electrophoresis device (Agilent, United States) with RNA 6000 Nano Assay Kit (Agilent, United States). Samples were of high quality (RNA Integrity Number >9) and purity (see Supplementary Table 1).

### Enzyme-Linked Immunosorbent Assay Test for Tumor Necrosis Factor α

Tumor Necrosis Factor α (TNF-α) concentration (SH NaCl *n* = 11, VG NaCl *n* = 10, SH LPS *n* = 11, VG LPS *n* = 12) was established as a control variable for confirming ongoing intraperitoneal inflammation. Plasma TNF-α concentration was determined using the Rat TNF-α ELISA Kit, from Diaclone, France (cat. no. 865.000), according to the manufacturer’s instructions. The same statistical assumptions and analytical procedure for HPLC data were used.

### High Performance Liquid Chromatography of Amino Acids

The concentration of the amino acid neurotransmitters: aspartate (ASP), glutamate (GLU), glycine (GLY), gamma-aminobutyric acid (GABA), tyrosine (TYR), and tryptophan (TRP) was determined in prepared HPT samples with RP-HPLC-ED gradient method with pre-column derivatization with fresh OPT-thiol reagent (0.1 M Borax, 0.5% OPT, 0.9% mercaptoethanol). The Agilent 1100 chromatographic system with Waters, AccQ-Tag for hydrolysate Amino Acid analysis chromatographic column (3.9 × 150 mm) preceded by a ZORBAX SB-C18 pre-column (4.6 × 12.5 mm) was used. The column temperature was set at 37°C and mobile phase flow at 0.4 ml/min. The carbon working electrode was set at +0.5 V, relative to the Ag/AgCl reference electrode. The gradient elution was performed using phosphate buffer (5.5 pH) containing: at 0.05 M NaH_2_PO_4_ × H_2_O, and addition of methanol; in buffer (A) 20% and (B) 80%. At the start, the mobile phase consisted of 100% buffer A. During the first 10 min of analysis, the concentration of buffer B was raised to 10% (in the 10th min: 90% A and 10% B). In the next 30 min, the content of buffer B in the mobile phase was raised from 10 to 85% (in 40th min: 15% A and 85% B). In the next 5 min, the content of buffer B was decreased to 0% (in the 45th min: 100% A and 0% B). After each sample, the system was re-equilibrated for 10 min. The chromatographic data were analyzed using ChemStation, Revision-B.03.02, Agilent software.

High performance liquid chromatography (HPLC) and TNF-α data were tested for normality (Shapiro–Wilk test) and homogeneity of variance (Levene’s test). Because some data did not meet the assumptions of the parametric test, we performed a Box-Cox transformation. Next, ANOVA with the Bonferroni *post hoc* test was used. Results of statistical tests and Box-Cox transformation formulas are presented Supplementary Table 5. The *p*-values lower than 0.05 were considered statistically significant. These statistical analyses were performed using the STATISTICA software, version 13.3 TIBCO Software Inc (2017).

### Microarray and Real-Time qPCR

Microarrays were used to analyze hypothalamic gene expression in all four experimental groups. The results of this analysis were validated using real-time qPCR.

### Microarray Preparation

A total of 21 samples (3–5 per experimental group, see Supplementary Table 1) were used for microarray analysis. These comprised the entire set of samples available in the experiment at that time. Samples were analyzed individually; 100 ng of the given sample that passed the initial quality control screen (2100 Bioanalyzer, Agilent) was labeled and amplified using GeneChip WT PLUS Reagent Kit and hybridized to an Affymetrix Rat Gene 2.1 ST Array Strip microarray using GeneAtlas Hybridization, Wash, and Stain Kit for WT Array Strips. GeneChip Poly-A RNA Control Kit and GeneChip Hybridization Controls were used to monitor the process of sample preparation. Hybridization to the microarrays was conducted for 20 h at 48°C. Following the hybridization, the microarrays were washed, stained, and scanned on a GeneAtlas™ System for Academic Customers. All the reagents and equipment for the microarray experiment were provided by Affymetrix, a subsidiary of Thermo Fisher Scientific, United States, and the procedures were performed according to the manufacturer’s protocol. The quality of the analyses was verified using Affymetrix Expression Console Software and standard Affymetrix quality metrics.

All Affymetrix.cel files were first imported into Partek^®^ Genomics Suite^®^ software, version 7, build 7.19.1125 (Partek, United States). Prior to any computations, the imported data set was normalized. Background correction was conducted with the use of the RMA method (Bolstad et al., 2003) and was followed by quantile normalization. Probes were logged using base 2, while the median polish (Mosteller and Tukey, 1977) algorithm was applied to probe set summarization. The definition of all contrasts of interest (comparisons of interest) constituted a base for the detection of differentially expressed genes, which was performed with the use of the ANOVA feature of Partek^®^ Genomics Suite^®^. As a result, every feature’s description contained (among others) *p*-value for a particular comparison accompanied by its FDR-corrected (Mosteller and Tukey, 1977) value and 95% confidence interval for those. The data set was annotated with the Ensembl Transcripts, release 100, genome assembly Rnor_6.0.

### qPCR Analysis

The results of the microarray analysis were validated using SYBR Green-based real-time qPCR. A total of 47 samples (including 21 samples used for microarray experiment, 8–11 samples per each experimental group, see Supplementary Table 1) were used for qPCR analysis. For each sample, 100 ng of total RNA was reverse-transcripted to cDNA using Smart RT-PCR Kit (EURx, Poland). Expression of 7 genes (*Actn2, Cxcl14, Gabrg1, Gria1, Oxt, Rab1b*, and *Vim*) was studied. Hmbs was used as a reference gene. It was identified as the most stably expressed out of a set of candidate reference genes (*Gapdh, Hmbs, Hprt1, Tbp*, and *Yhwaz*) using the NormFinder tool (see Supplementary Table 3). For details on the method of selecting the reference gene, see our previous work (Stankiewicz et al., 2015). For detailed primers description, see Supplementary Table 2. Rotor-Gene Q (Qiagen, Netherlands) with Fast SG qPCR Master Mix (2x) (EURx, Poland) were used to perform qPCR. qPCR reaction conditions were as follows: (1) denaturation −95°C for 50 s; (2) amplification, 35 cycles – 95°C for 10 s then 10 s in primer-specific annealing temperature then 72°C for 15 s; (3) melting curve analysis – 72–95°C. All assays were executed in triplicate. Each run included five 4-fold serial dilutions for calculating reaction efficiency. A melting curve analysis was performed to verify the presence of one gene-specific peak and the absence of primer-dimer peaks. The Pfaffl model was used to calculate relative expression ratios of studied genes. Detailed data on qPCR analysis are presented in Supplementary Table 2. qPCR was performed according to the Minimum Information for Publication of Quantitative Real-Time PCR Experiments (MIQE) guidelines (Bustin et al., 2009). Shapiro–Wilk test was used to test the normality of qPCR data. As the data were not distributed normally, Mann–Whitney–Wilcoxon test was used to investigate the significance of comparisons, which showed a non-adjusted *p*-value lower than 0.05 in the microarray experiment. As multiple comparisons were made within each analyzed gene, the Benjamini-Hochberg method (Benjamini and Hochberg, 1995) was used to control False Discovery Rate. The R script used for statistical analysis of the results can be found in Supplementary Presentation 1.

## Results

### TNF-α-Based Validation of Inflammatory Response

**FIGURE 1:**
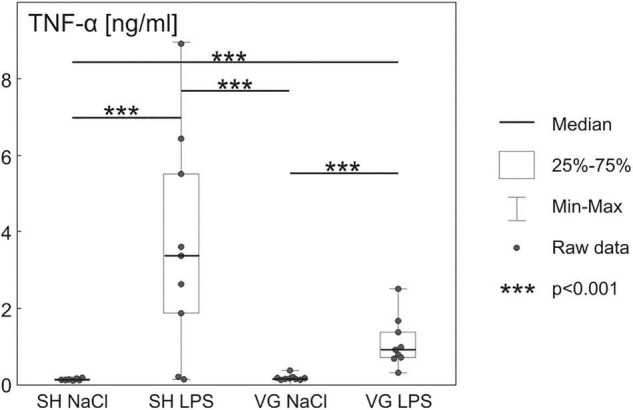
Increased plasma tumor necrosis factor α (TNF-α) concentration after intraperitoneal injection of LPS confirm initiation of pro-inflammatory activity of the immune system. Details in text.

Increased plasma TNF-α (Figure 1) concentration confirms the development of inflammatory conditions in both rat groups injected with LPS [*F*(3,34) = 28.191, *p* < 0.001, SH NaCl vs. SH LPS *p* < 0.001, VG NaCl vs. VG LPS *p* < 0.001]. However, the inflammatory response of vagotomized animals seems to be stunted in comparison to sham control, which is in line with observations made by Ghia et al. (2007).

### High Performance Liquid Chromatography Analysis

**FIGURE 2:**
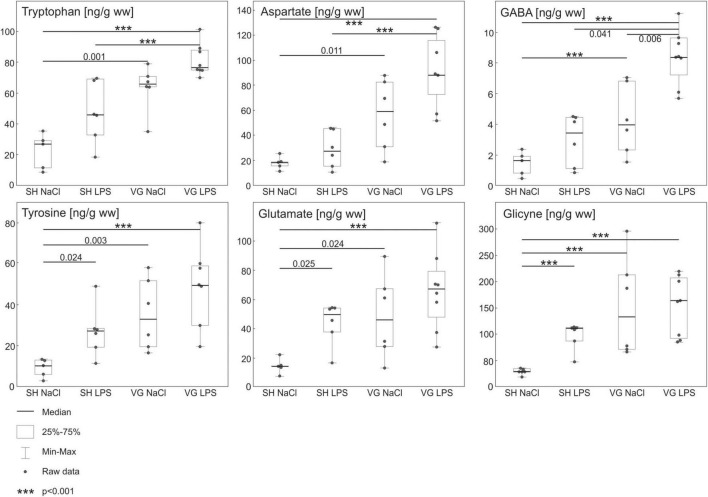
Results of high performance liquid chromatography (HPLC) analysis of amino acids in hypothalamus. Concentrations of monoaminergic neurotransmitter precursors (Tyrosine, Tryptophan) and inhibitory amino acid neurotransmitters (GABA, Glycine) are increased in vagotomized group (VG NaCl vs. SH NaCl). Similar effect is absent in case of excitatory amino acid neurotransmitters (Glutamate, Aspartate). During intraperitoneal inflammation changes in tyrosine and tryptophan concentrations in vagotomized group (VG LPS vs. SH LPS) are even more pronounced. Additionally, we observe a significant increase of inhibitory GABA and excitatory aspartate concentration in that group (VG LPS vs. SH LPS). Details in text.

Statistical analysis revealed significant differences between groups in concentrations of all measured hypothalamic amino acids concentration (Figure 2) [TRP *F*(3,21) = 18.292, *p* < 0.001, TYR *F*(3,21) = 10.419, *p* < 0.001, ASP *F*(3,21) = 15.473, *p* < 0.001, GLU *F*(3,21) = 8.019, *p* < 0.001, GABA *F*(3,21) = 18.020, *p* < 0.001, GLY *F*(3,21) = 20.72, *p* < 0.001]. Intraperitoneal injection of LPS did not affect hypothalamic TRP, ASP, and GABA, concentrations in SH group (SH NaCl vs. SH LPS: TRP *p* = 0.077, ASP *p* = 1, GABA *p* = 0.604). Subdiaphragmatic vagotomy significantly increased the concentration of these amino acids despite LPS conditions (SH NaCl vs. VG NaCl: TRP *p* = 0.001, ASP *p* = 0.011, GABA *p* < 0.001; SH LPS vs. VG LPS: TRP *p* < 0.001, ASP *p* < 0.001, GABA *p* = 0.041). Worth noting, GABA concentration in VG group is increased after LPS administration (VG NaCl vs. VG LPS *p* = 0.006). On the other hand, intraperitoneal injection of LPS significantly increased hypothalamic TYR, GLY, and GLU concentrations in SH group (SH NaCl vs. SH LPS: TYR *p* = 0.024, GLU *p* = 0.025, GLY *p* < 0.001). Subdiaphragmatic vagotomy significantly increased TYR, GLY, and GLU concentrations in non-septic conditions (SH NaCl vs. VG NaCl: TYR *p* = 0.003, GLU *p* = 0.024, GLY *p* < 0.001). Also, after LPS administration, TYR, GLY, and GLU concentrations are elevated in similar manner as VG NaCl group – significant increased compare to SH NaCl group conditions (SH NaCl vs. VG LPS: TYR *p* < 0.001, GLU *p* < 0.001, GLY *p* < 0.001) and no differences between vagotomized groups (VG NaCl vs. VG LPS: TYR *p* = 1, GLU *p* = 0.886, GLY *p* = 1). Therefore, no significant differences between SH LPS and VG LPS are observed (SH LPS vs. VG LPS: TYR *p* = 0.262, GLU *p* = 0.840, GLY *p* = 0.619).

### Microarray Experiment

**FIGURE 3:**
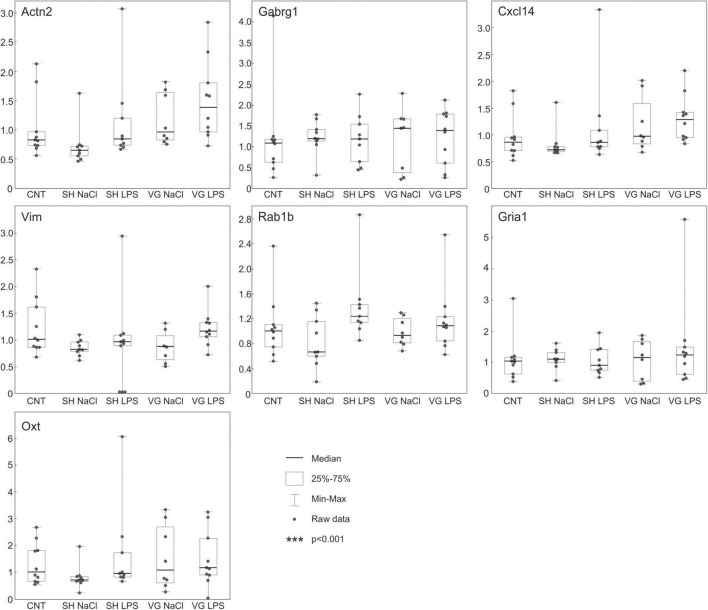
QPCR analysis confirms that experimental procedures did not affect the hypothalamic gene expression pattern. The whiskers extend from the hinge to the smallest/largest value no further than 1.5 × IQR from the hinge (where IQR is the inter-quartile range, or distance between the first and third quartiles). Data beyond the end of the whiskers are called “outlying” points and are plotted individually.

Using microarrays, we have analyzed transcriptomic patterns induced by experimental conditions in the hypothalamus. None of the observed statistically significant changes in expression of analyzed transcripts survived adjustment for multiple comparisons. Nevertheless, we have selected 7 genes characterized by relatively low non-adjusted *p*-values in some of the comparisons (mean = 0.019, SD = 0.01, also see Supplementary Table 4), and analyzed their expression using qPCR (Figure 3). The qPCR analysis showed that the studied genes (*Actn2, Cxcl14, Gabrg1, Gria1, Oxt, Rab1b*, and *Vim*) do not respond to experimental conditions, thus supporting data from microarrays (see Supplementary Table 4).

## Discussion

### Vagotomy Affects Precursors of Monoamine Neurotransmitters in the Hypothalamus

Vagal denervation attenuates inflammation-induced increase of plasma corticosterone and ACTH concentrations for up to 2 weeks (Fleshner et al., 1995; Wieczorek and Dunn, 2006). Afterward, this effect of vagotomy is diminished. This indicates that the proper functioning of the anti-inflammatory HPA axis is restored in the long run.

However, even after 1 month following a vagotomy, a monoaminergic neurotransmission in the hypothalamus (a first element of the anti-inflammatory HPA axis), as well as in other limbic system structures, is deregulated (Kobrzycka et al., 2019). We confirmed these results in this work by observing an increase in a number of precursors of the monoaminergic neurotransmitters (tyrosine and tryptophan) in the hypothalamus of the vagotomized animals. In fact, a similar tendency to increase tryptophan concentration was also noticed by Wieczorek and Dunn (2006) in many brain areas of vagotomized mice. CNS may require higher amounts of the precursors because they are necessary for reported increased dopamine and serotonin synthesis and turnover in many brain regions after the subdiaphragmatic vagotomy (Wieczorek and Dunn, 2006; Kobrzycka et al., 2019). It is also worth noting that one of the side effects of subdiaphragmatic vagotomy procedure is a slowed movement of food through the digestive system (Martin et al., 1977), which also may affect the content of tyrosine and tryptophan in blood (Fernstrom and Fernstrom, 2007; Kapalka, 2010; Al Mushref and Srinivasan, 2013; Fu et al., 2014).

### Vagotomy Affects Amino Acid Neurotransmitters in the Hypothalamus

Hypothalamus is innervated by monoaminergic fibers originating from a number of brain areas directly or indirectly affected by nucleus tractus solitarius (NTS), which distributes the sensory information from the vagus nerve (Rinaman, 2007; Myers et al., 2012; Elson and Simerly, 2015; Miller and Yeh, 2017; Weidenfeld and Ovadia, 2017). Those innervations modulate hypothalamic functions, i.e., by affecting hypothalamic concentrations of amino acid neurotransmitters (e.g., GABA and Glycine) and, subsequently, post-synaptic potentials (Miller and Yeh, 2017). Previously, we observed vagotomy caused changes in monoaminergic neurotransmission in many of those areas (e.g., AM, PAG, and HIP) and the hypothalamus itself. In this work, we show a modest increase in the hypothalamic concentration of amino acid neurotransmitters in full-recovered animals subjected previously to vagotomy as compared to sham procedure. Similar results were reported by Klarer et al. (2014), who studied amino acid neurotransmitters in several other limbic brain structures. Based on these results, we hypothesize that those vagotomy-induced changes in hypothalamic amino acid neurotransmitters may be caused by deregulated monoaminergic signaling originating in limbic structures.

Glutamate, aspartate, and glycine innervation of the hypothalamus originate from the brainstem and internal hypothalamic circuit (Ziegler et al., 2005; Noori et al., 2017; Varga et al., 2019). Glutamate, aspartate, and glycine inhibit CRH release from the hypothalamus *in vitro* (Buckingham and Hodges, 1977; Patchev et al., 1994). In our experiment, we observed increased levels of all of these neurotransmitters in the vagotomized group regardless of LPS-induced inflammation. Such observation suggests suppression of CRH release and in consequence HPA axis activity. However, such a hypothesis would be in opposition to the previously mentioned restoration of LPS-induced corticosterone increase in long-term recovered vagotomized rats. This apparent incompatibility might be explained by intensified GABA concentration in the hypothalamus.

In basal conditions, the tonic, inter-hypothalamic GABA circuit suppresses the activity of hypothalamic CRH-releasing neurons (Kovács et al., 2004). Our results show that this mechanism is intensified in vagotomized animals compared to sham-operated animals. We propose that this intensification of basal hypothalamic GABA release may underlie reported down-regulation of corticosterone release in animals that underwent vagotomy procedure a few days before measurement (Fleshner et al., 1995; Wieczorek and Dunn, 2006). As we report here, 30 days after vagotomy procedure, hypothalamic GABA concentration is even higher in inflammatory conditions. We think that this may be a result of enhanced external inhibitory signals, especially amygdalic GABA-GABA disinhibitory connections (Herman et al., 2004), which would inhibit the activity of the inter-hypothalamic GABA circuits. Thus, the internal hypothalamic inhibition of CRH release would be suppressed, and in consequence, hypothalamic hormonal activity and further adrenal corticosterone release in response to inflammation would be the same as in sham animals (Kobrzycka et al., 2019). This is indeed what we observe in our studies. However, this hypothesis requires further, more detailed investigation.

Another amino acid crucial for hypothalamic functioning is glycine. Glycine inhibits orexin- mediated arousal, energy homeostasis, and reward-seeking. It also promotes sleep and decreases body temperature during sleep episodes *via* peripheral vasodilation (Kawai et al., 2015). In conscious rats, glycine acts in PVN to enhance the renal excretion of water and sodium and decrease central sympathetic outflow to the heart and kidneys (Krowicki and Kapusta, 2011). Glycine can act allosterically as an excitatory modulator of the NMDA subtype of ionotropic glutamate receptors and through the activation of NMDA receptors in suprachiasmatic nucleus (SCN). Glycine is also involved in oxytocin and vasopressin synthesis in magnocellular neurosecretory cells (MNCs) in the PVN and SON (supraoptic nucleus) and a significant tonic inhibitory effect is mediated through ionotropic GlyR (Choe et al., 2016).

In the context of HPA axis activity, glycine can inhibit hypothalamic vasopressin synthesis (Hussy et al., 2001; Choe et al., 2016) which synergistically to CRH stimulates ACTH secretion (Mazzocchi et al., 1997; Papadimitriou and Priftis, 2009). Glycine also decreased the spontaneous release of CRH from the hypothalamus but does not affect hypothalamic CRH content (Buckingham and Hodges, 1977). This observation comes, however, from a study carried out on isolated hypothalamic tissue with restrictive controlled neurotransmission alterations – that does not consider simultaneously changes of other neurotransmitters content. *In vivo* studies showed that intracerebroventricular (Rundgren et al., 1993) or intravenous (Hahn et al., 1991) glycine infusion may increase vasopressin plasma levels. This difference originating from the different routes of glycine administration might be an important factor in the interpretation of our results.

The raphe magnus and the ventrolateral periaqueductal gray were found to be the exclusive sources of the inhibitory glycinergic innervation in the PVN (Varga et al., 2019). Although sufficient amounts of glycine are synthesized *de novo*, exogenous glycine passively diffuses across the blood-brain barrier and modulates neurotransmission in the CNS (Kawai et al., 2015). Observed after vagotomy increase in hypothalamic glycine concentration might result from altered neurotransmission of other brain structures, but also, as mentioned before, from altered after vagotomy peristalsis.

We do not know how the immune signals reach the CNS in vagotomy conditions in the early stages of inflammation. We think that this may be an effect of an alternative mechanism replacing disrupted vagus-mediated signaling pathway. Wieczorek and Dunn (2006) suggested that this mechanism might be associated with changes in prostaglandin signaling. In fact, Dalli et al. (2017) showed that even unilateral subdiaphragmatic truncal vagotomy significantly alters macrophage lipid profile, which is involved in the metabolism of arachidonic acid during intraperitoneal inflammation. Additionally, they reported that following left trunk vagotomy, the expression of COX2, an enzyme involved in PGE2 synthesis, significantly increased in macrophages (Dalli et al., 2017). We consider that elevation of inflammation-induced PGE2 synthesis may take over immune-to-CNS communication after vagotomy. We think that elevation of inflammation-induced PGE2 synthesis may take over immune-to-CNS communication after vagotomy, as we suggested previously (Kobrzycka et al., 2019). In theory, blood-borne PGE2 reaches the brain *via* the bloodstream, where it acts upon epithelial cells of blood vessels. These vessels preserve intact prostaglandin-related cellular machinery after vagotomy (Sergeev and Akmaev, 2000). Once at the brain, PGE2 activates CVOs, PVN, and NTS (Sekiyama et al., 1995; Ek et al., 1998; Rocca and FitzGerald, 2002; Cai et al., 2014; Le Maître et al., 2015) and subsequently, other brain areas in a way similar to the now non-functional vagus nerve (Sekiyama et al., 1995; Marty et al., 2008).

Some authors proposed an alternative to the vagal, neural route of immune to CNS communication. Splanchnic sympathetic nerves were shown to control inflammation processes, induced by intravenous administration of LPS (Martelli et al., 2014, 2019). It was proposed that splanchnic sympathetic nerves constitute an efferent part of the inflammatory reflex along with vagal afferents or/and bloodstream as sensory routes of immune to CNS communication (Komegae et al., 2018; McAllen et al., 2022). We cannot exclude that after abdominal vagal denervation, part of immune sensory functions is taken over by other sensory fibers. However, we could not find information about such a process occurring during intraperitoneal inflammation. Until such a process is observed, the vagus nerve is still considered the main immune sensory neural pathway from the abdominal cavity to CNS (Pavlov and Tracey, 2017).

Microarray and subsequent Real-Time qPCR analysis suggest that the transcriptome does not affect the activity of the hypothalamus during inflammation in vagotomized rats after a 30-day-long recovery period. It would be interesting to see whether gene expression changes are more pronounced at earlier stages of recovery while later giving way to other compensatory mechanisms such as neurotransmission alterations. On the other hand, we found that amino acid neurotransmitters are deregulated in the hypothalamus of vagotomized animals after prolonged recovery. This, along with our previous finding on deregulated monoamine systems in brain structures affecting the functioning of the hypothalamus, suggests that vagal role in the regulation of HPA axis after a longer recovery period is replaced by changes in external neurotransmitter modulation of hypothalamic activity rather than alteration of basic hypothalamic functionality. Of course, the presented data do not fully describe the observed phenomena of restored HPA axis activity in recovered vagotomized animals. Previously, we presented changes in CNS monoaminergic neurotransmission. Here, we report changes in amino acid components in the hypothalamus. In neuronal tissue, amino acid neurotransmitters play a critical role in electrophysiological processes not investigated in this study. We also pay attention that observed changes might be evolving in time along with prolonged recovery after vagotomy. For future studies, we would like to extend our research to a few time points that allow us to establish the time dynamic of presented processes. For now, we simply report that hypothalamic amino acid neurotransmission is altered 30 days after vagotomy and it is an interesting issue for discussion and further investigation.

## Data Availability Statement

The datasets presented in this study can be found in online repositories. The names of the repository/repositories and accession number(s) can be found below: https://www.ncbi.nlm.nih.gov/geo/query/acc.cgi?acc=GSE199231.

## Ethics Statement

The animal study was reviewed and approved by the Local Ethical Committee for Animal Experiments in Łodz, 73/ŁB582/2012.

## Author Contributions

AK wrote the manuscript, isolated material for the microarray and qPCR analyses, and performed the qPCR and HPLC analysis as well as statistical analysis of the HPLC and ELISA results. AS wrote the manuscript, and oversaw and participated in isolation of the material for the microarray and qPCR analyses and coordinated them. MW designed the experiment, performed the experimental procedures on living animals, and collected the samples. JG performed the bioinformatic and statistical analyses of microarray data. MG, BB, and RI-N performed the microarray analysis. KP-K performed the ELISA tests. All authors contributed to the article and approved the submitted version.

## Conflict of Interest

The authors declare that the research was conducted in the absence of any commercial or financial relationships that could be construed as a potential conflict of interest.

## Publisher’s Note

All claims expressed in this article are solely those of the authors and do not necessarily represent those of their affiliated organizations, or those of the publisher, the editors and the reviewers. Any product that may be evaluated in this article, or claim that may be made by its manufacturer, is not guaranteed or endorsed by the publisher.
