# Supplementary material for: Hypothalamic Neurochemical Changes in Long-Term Recovered Bilateral Subdiaphragmatic Vagotomized Rats
Source: Front Behav Neurosci. 2022 Jul 8;16:869526. doi: 10.3389/fnbeh.2022.869526 (PMC9304976; doi:10.3389/fnbeh.2022.869526)
Supplement: Supplementary file 2 [file Presentation_1.pdf]

Supplementary Presentation 1: The R script used for statistical analysis of the QPCR results

```
library(tidyverse)

qpcrResults <- readr::read_tsv("qpcr_results.tsv")

tidy_qpcrResults <- qpcrResults %>%
  tidyr::gather(key = "Gene", value = "ratio (R)", -c(Group, Name) )

tidy_qpcrResults %>%
  mutate(Group = recode(Group, C = "Control", S_1 = "Sham+LPS", S_3 = "Sham+NaCl",
V_1 = "Vagotomy+LPS", V_3 = "Vagotomy+NaCl")) %>%
  ggplot(aes(x = Group, y = `ratio (R)`, fill = Group))+
  geom_boxplot()+
  facet_wrap(~Gene)+
  theme_light()

### Lets make Mann-Whitney-Wilcoxon
#wilcox.test(formula, data, subset) - needs 2 levels of factor for comparison

MWW_test <- wilcox.test(Actn2 ~ Group, data = qpcrResults, subset = Group == c("C",
"S_1"))

MWW_test <- qpcrResults

MWW_test$C_vs_S_1 <-   lapply(X = qpcrResults[3:9], FUN = function(X) { wilcox.test(X
~ qpcrResults$Group, subset = qpcrResults$Group == c("C", "S_1")) })
```

```
MWW_test$C_vs_S_3 <-   lapply(X = qpcrResults[3:9], FUN = function(X) { wilcox.test(X ~ qpcrResults$Group, subset = qpcrResults$Group == c("C", "S_3")) })

MWW_test$C_vs_V_1 <-   lapply(X = qpcrResults[3:9], FUN = function(X) { wilcox.test(X ~ qpcrResults$Group, subset = qpcrResults$Group == c("C", "V_1")) })

MWW_test$C_vs_V_3 <-   lapply(X = qpcrResults[3:9], FUN = function(X) { wilcox.test(X ~ qpcrResults$Group, subset = qpcrResults$Group == c("C", "V_3")) })

MWW_test$S_1_vs_S_3 <-   lapply(X = qpcrResults[3:9], FUN = function(X) { wilcox.test(X ~ qpcrResults$Group, subset = qpcrResults$Group == c("S_1", "S_3")) })

MWW_test$S_1_vs_V_1 <-   lapply(X = qpcrResults[3:9], FUN = function(X) { wilcox.test(X ~ qpcrResults$Group, subset = qpcrResults$Group == c("S_1", "V_1")) })

MWW_test$S_3_vs_V_3 <-   lapply(X = qpcrResults[3:9], FUN = function(X) { wilcox.test(X ~ qpcrResults$Group, subset = qpcrResults$Group == c("S_3", "V_3")) })

MWW_test$V_1_vs_V_3 <-   lapply(X = qpcrResults[3:9], FUN = function(X) { wilcox.test(X ~ qpcrResults$Group, subset = qpcrResults$Group == c("V_1", "V_3")) })

# Write-out resulting analysis
write_lines(x = rjson::toJSON(MWW_test), path = "MWW_test.json")
### Lets make Mann-Whitney-Wilcoxon



### Lets make a nice list o pvalues from Mann-Whitney-Wilcoxon
MWW_pval <- list()
nb <- 1
for(n_comp in seq_along(MWW_test))
  {
```

```
  for(n_gene in seq_along(MWW_test[[n_comp]]))
    {
    MWW_pval$Comparison[[nb]] <- names(MWW_test[n_comp])
    MWW_pval$Gene[[nb]] <- names(MWW_test[[n_comp]][n_gene])
    MWW_pval$pval[[nb]] <- MWW_test[[n_comp]][[n_gene]]$p.value
    nb = nb + 1
    }

  }

df_MWW_pval <- as_tibble(MWW_pval)
### Lets make a nice list o pvalues from Mann-Whitney-Wilcoxon




### Lets filter in only comparisons/genes, that were significant in MA
which_comparisons_were_significant <-
readr::read_tsv("which_comparisons_were_significant.txt")

tidy_which_comparisons_were_significant <- which_comparisons_were_significant %>%
  tidyr::gather(key = "Gene", value = "ma", -c(Comparison) ) %>%
  mutate(
    key = paste0(Comparison, Gene),
    Comparison = NULL,
    Gene = NULL
  )

forMerge_df_MWW_pval <- df_MWW_pval %>%
  mutate(
    key = paste0(Comparison, Gene)
```

```
  )

merged_forMerge_df_MWW_pval <- merge(forMerge_df_MWW_pval, tidy_which_comparisons_were_significant, by = "key") %>%
  select(-key) %>%
  filter(!is.na(ma)) %>%
  select(-ma)
### Lets filter in only comparisons/genes, that were significant in MA




### Lets look at means and SDs
meanSD_tidy_qpcrResults <- tidy_qpcrResults %>%
  select(-Name) %>%
  group_by(Group, Gene) %>%
  nest() %>%
  mutate(
    Srednia = data %>% map_dbl(map_dbl, mean),
    Od_std = data %>% map_dbl(~.[[1]] %>% sd)) %>% ## THIS WILL BE MY MAPPING METHOD OF CHOICE FOR NOW
  unnest()




### Here we make SW test for normality
SW_tidy_qpcrResults <- tidy_qpcrResults %>%
  select(-Name) %>%
  dplyr::group_by(Gene, Group) %>%
  nest()
```

```
library(magrittr)

SW_results <- SW_tidy_qpcrResults %$%
  map(data, ~ shapiro.test(.x[[1]]))


SW_tidy_qpcrResults <- SW_tidy_qpcrResults %>%
  dplyr::mutate(Shapiro_Wilk_p_val =
                map_chr(SW_results, ~ .x$p.value )
  )




### Here we find out whether any SW analysis gave significant value, meaning that distribution of Cqs in given group in given gene is not normal.

is_gene_normal <- SW_tidy_qpcrResults %>%
  select( -c(data, Group) ) %>%
  group_by(Gene) %>%
  nest() %>%
  mutate( gene_is_normal = data %>% map(~ case_when(
    .x < 0.05  ~ FALSE,
    TRUE ~ TRUE
  ) ) ) %>%
  mutate( gene_is_normal_short =
          gene_is_normal %>% map( ~ !any(.x) )
  )




### Lets statistically adjust Mann-Whitney-Wilcoxon tests

geneList_merged_forMerge_df_MWW_pval <- merged_forMerge_df_MWW_pval %>%
  group_by(Gene) %>%
  nest()
```

```
geneList_merged_forMerge_df_MWW_pval$data <- map(.x =
geneList_merged_forMerge_df_MWW_pval$data, .f =

    ~ mutate(.data = .x, MWW_adjusted = p.adjust(as.numeric(.x$pval), method = "BH")

    )

)


geneList_merged_forMerge_df_MWW_pval <- geneList_merged_forMerge_df_MWW_pval
%>%

  unnest()



write_tsv(x = geneList_merged_forMerge_df_MWW_pval, path =
"adjusted_MWW_pval.tsv")

### Lets statistically adjust Mann-Whitney-Wilcoxon tests
```
